# Serum LL‐37 and inflammatory cytokines levels in psoriasis

**DOI:** 10.1002/iid3.802

**Published:** 2023-03-14

**Authors:** Juanfeng Lao, Zhi Xie, Qunshi Qin, Ru Qin, Shangyang Li, Yulin Yuan

**Affiliations:** ^1^ Department of Laboratory Medicine, Guangxi Academy of Medical Sciences The People's Hospital of Guangxi Zhuang Autonomous Region Nanning Guangxi China; ^2^ Department of Dermatology and Venereology, Guangxi Academy of Medical Sciences The People's Hospital of Guangxi Zhuang Autonomous Region Nanning Guangxi China

**Keywords:** inflammatory cytokines, LL‐37, psoriasis

## Abstract

**Background:**

Psoriasis (PsO) is a T‐cell‐associated inflammatory autoimmune dermatitis. Leucine leucine‐37 (LL‐37) is upregulated in PsO patients and correlated with the area and severity of PsO. However, the exact relation between LL‐37 and T cell‐associated inflammation is not well understood. It is very important to clarify the relationship between LL‐37 and inflammatory response for clinical diagnosis and treatment of PsO. This study investigated the serum levels of LL‐37 and inflammatory cytokines, as well as correlations between them in PsO patients, which aimed to provide new ideas for the diagnosis and treatment of PsO.

**Methods:**

PsO patients (*n* = 50) and healthy volunteers (*n* = 33) were recruited in this study. Skin specimens were stained with hematoxylin and eosin (H&E). The serum levels of LL‐37, T‐helper type 1 (Th1, IFN‐γ), T‐helper type 17 (Th17, IL‐17), T‐helper type 22 (Th22, IL‐22), and T‐helper type 2 cytokines (Th2, IL‐4) were assessed by enzyme‐linked immunosorbent assay. Some of the patients were re‐recruited after treatment to evaluate LL‐37 and cytokines levels.

**Results:**

Pathological changes were observed in PsO skin lesions. LL‐37, IFN‐γ, IL‐17, and IL‐22 serum levels were much higher in PsO patients than those in healthy volunteers (*p* < .001), and posttreatment reduction was observed in five patients. However, no remarkable difference in IL‐4 level (*p* > .05) was found. LL‐37 level was positively correlated with IFN‐γ, IL‐17, and IL‐22 levels (*p* < .001) in PsO patients.

**Conclusion:**

LL‐37 expression was significantly associated with inflammatory response, which may provide us new ideas for diagnosing and monitoring disease activity of PsO.

## Graphical abstract

Leucine leucine‐37 (LL‐37), IFN‐γ, IL‐17, and IL‐22 serum levels were much higher in PsO patients than those in healthy volunteers, and posttreatment reduction was observed. LL‐37 level was positively correlated with IFN‐γ, IL‐17, and IL‐22 levels in PsO patients. These results indicated that LL‐37 may regulate PsO pathogenesis by modulating in Th1, Th17, and Th22 responses.

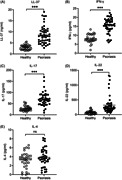

## 1 INTRODUCTION

Psoriasis (PsO) is a chronic inflammatory skin disorder characterized by sharply demarcated scaly erythematous plaques which might spread to giant body areas.^1^, ^2^ T lymphocytes function as key players in PsO pathogenesis.^3^, ^4^ In psoriatic skin lesions, T cells stimulate excessive proliferation of keratinocytes, neutrophil granulocytes recruitment and activation, and secretion of inflammatory mediators.^4^, ^5^ The PsO pathogenesis is mainly driven by Th1, Th17, and Th22, resulting aberrant release of corresponding inflammatory cytokines, including IFN‐γ, TNF‐α, IL‐12, IL‐17, and IL‐22.^3^, ^6^, ^7^ The unbalanced interactions among skin resident cells, innate immune cells (neutrophils and DCs), and adaptive immune cells contribute to pathological features of psoriatic inflammation such as acanthosis, hyperkeratosis, and parakeratosis.^8^, ^9^ The etiology of PsO is complex, and the specific mechanism needs to be further investigated.

Recently, diagnosis of PsO mainly depends on the assessment of medical history and clinical signs by trained dermatologists, which requires the collaboration of a specialized and trained dermatologist, laboratory technician, and dermatopathologist. To better diagnose and treat PsO, a wide range of biomarkers and targets have been investigated.^10^, ^11^, ^12^ Inflammatory‐related mediators such as IL‐17A, IL23A, and TNF‐α have been reported to be important biomarkers for disease activity of PsO.^2^, ^10^ Based on the pathological process of PsO, targeting related signaling pathways and molecules can be an effective way to develop therapeutic drugs. In an imiquimod (IMQ)‐induced mouse model, Bruton's tyrosine kinase inhibitors significantly reduced psoriatic inflammation.^13^ Inhibition of the JAK/STAT3 pathway to reduce Th17/Treg ratio and promoting M2 polarization contributed to alleviating PsO.^14^ At present, biological therapies targeting IL‐12/IL‐23, TNF‐α, and IL‐17 have been approved for moderate to severe PsO treatments.^15^, ^16^ In addition, Th2‐induced cytokines, namely IL‐4 and IL‐10, have been reported to be promising targets for PsO.^17^, ^18^, ^19^, ^20^ Since PsO is an organ‐specific T cell‐dependent inflammatory disorder, specifically targeting relevant cytokines to the inflammatory pathway can be an effective treatment option for PsO.

Leucine leucine‐37 (LL‐37), an antimicrobial peptide, originates from the cathelicidin hCAP‐18, which is a human cationic peptide and expressed by macrophages, lymphocytes, neutrophils, and epithelial cells.^21^, ^22^, ^23^ Our previous data revealed that circulating LL‐37 level was markedly increased in PsO patients, which could be a promising diagnostic and therapeutic biomarker in PsO.^24^ It has been reported that LL‐37 is elevated in psoriatic keratinocytes.^25^ LL‐37 triggers plasmacytoid DCs activation and induces IFN‐α secretion through LL‐37‐DNA complex formation.^25^ IFN‐α secretion stimulates pathogenic T cells activation, leading to aberrant Th1/Th17 differentiation and keratinocyte proliferation, as well as the formation of psoriatic lesions.^26^, ^27^ Circulating T cells from PsO patients also recognize LL‐37 as an autoantigen, thereby producing IL‐17 and IFN‐γ.^28^ Previous studies have found that LL‐37 level on skin lesions is closely related with proinflammatory cytokines production in PsO patients.^29^ Nonetheless, the crosstalk between circulating levels of LL‐37 and inflammatory cytokines remains ambiguous.

In the present study, LL‐37 and inflammatory cytokines serum levels were examined in PsO patients and healthy volunteers, and analyzed by correlation method. Our results indicated that circulating levels of LL‐37, IFN‐γ, IL‐17, and IL‐22 were markedly elevated in PsO patients, and posttreatment reduction was observed. In addition, serum LL‐37 level was positively correlated with these proinflammatory cytokines. Our study indicated that LL‐37 may regulate PsO pathogenesis by modulating in Th1, Th17, and Th22 responses, which provided us new ideas for the diagnosis and treatment of PsO.

## 2 MATERIALS AND METHODS

### 2.1 Human subjects

Fifty PsO patients from the Department of Dermatology and Venereology in The People's Hospital of Guangxi Zhuang Autonomous Region were diagnosed with PsO from September 2021 to February 2022. Patients with chronic‐inflammatory, allergic, autoimmunological, metabolic, neoplastic diseases, or any other diseases that may affect the outcome of PsO were excluded. This study recruited 33 healthy volunteers without PsO or any family history of PsO. These healthy donors were from the Health Examination Center of The People's Hospital of Guangxi Zhuang Autonomous Region between September 2021 to February 2022 (Supporting Information: Table 1). The control group was age‐ and sex‐matched.

### 2.2 Skin biopsy collection and histological analysis

Skin biopsies were dissected from a 3 cm psoriatic lesion by dermatologists and diagnosed as lesional psoriatic skin. Normal skin biopsies were collected from healthy volunteers in a similar body area to psoriatic skin by the same method. Skin tissues were subjected to fixation, embedding and sectioning (5 µm), and stained with hematoxylin and eosin (H&E) as previously described.^30^ Images were assessed under a microscope (Olympus BX53).

### 2.3 Enzyme‐linked immunosorbent assay (ELISA) assay

Venous blood samples (5 mL) were collected by venipuncture without anticoagulant under sterile conditions from all PsO patients and healthy volunteers. Within 30 min, samples were rapidly centrifuged for 5 min at 1500 rpm/min and then serum samples were collected and stored at −80°C immediately until the assays were conducted. LL‐37, IFN‐γ, IL‐22, IL‐17, and IL‐4 levels were quantified using commercial ELISA Kit (FineTest). All assays were conducted according to manufacturer's protocols.

### 2.4 Statistical analysis

Data analyses were conducted using Prism 8.0 Software. The Mann−Whitney test was used to evaluate difference between groups, and Pearson's correlation test was employed for correlation analysis. *p* < .05 was considered statistically significant.

## 3 RESULTS

### 3.1 Clinical and histological characteristics of PsO

**Figure 1:**
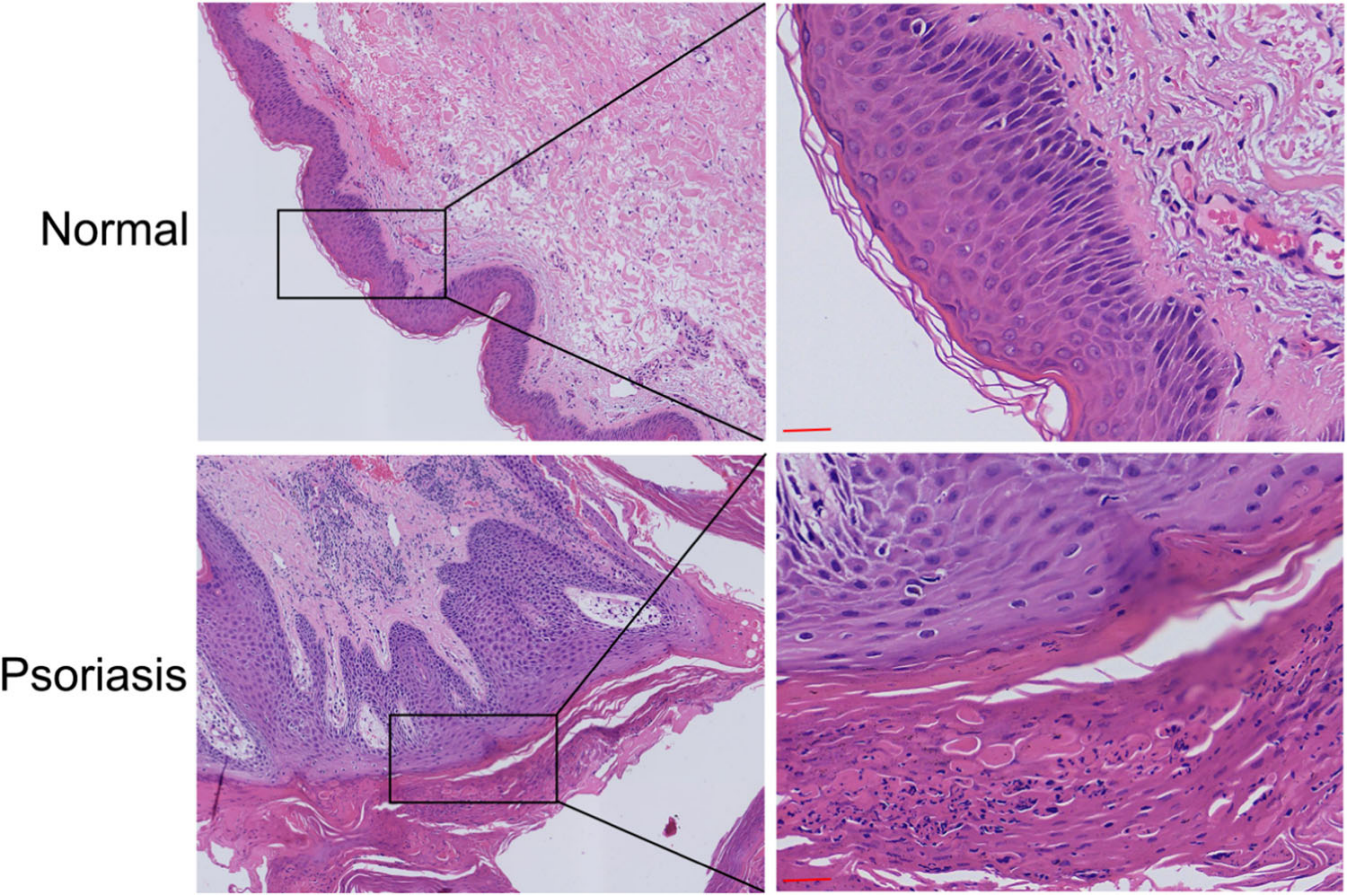
Representative images of H&E stained normal and psoriatic skin biopsies. Formalin‐fixed paraffin‐embedded sections were stained with H&E. Scale bar = 30 µm. H&E, hematoxylin and eosin.

A cohort of 50 PsO patients were recruited in this study. Clinical characteristics are listed in Supporting Information: Table 1. Representative images of normal and PsO lesional skin sections stained with H&E were shown in Figure 1. Compared with the normal counterparts, psoriatic skin displayed extensive hyperkeratosis with fusional keratosis. The epidermis appeared hyperplasia and grew downwards with the similar length. The granular layer became thinner or disappeared locally. The spinous layer was slightly spongy. Dermal papillae appeared edema and blood vessels became dilated, along with the upper epidermis thinning. Lymphocyte infiltration was observed around the shallow blood vessels. Changes in the epidermis of skin in PsO patients may contribute to the impairment of their barrier functions.

### 3.2 Increased serum LL‐37 and proinflammatory cytokines levels in PsO

**Figure 2:**
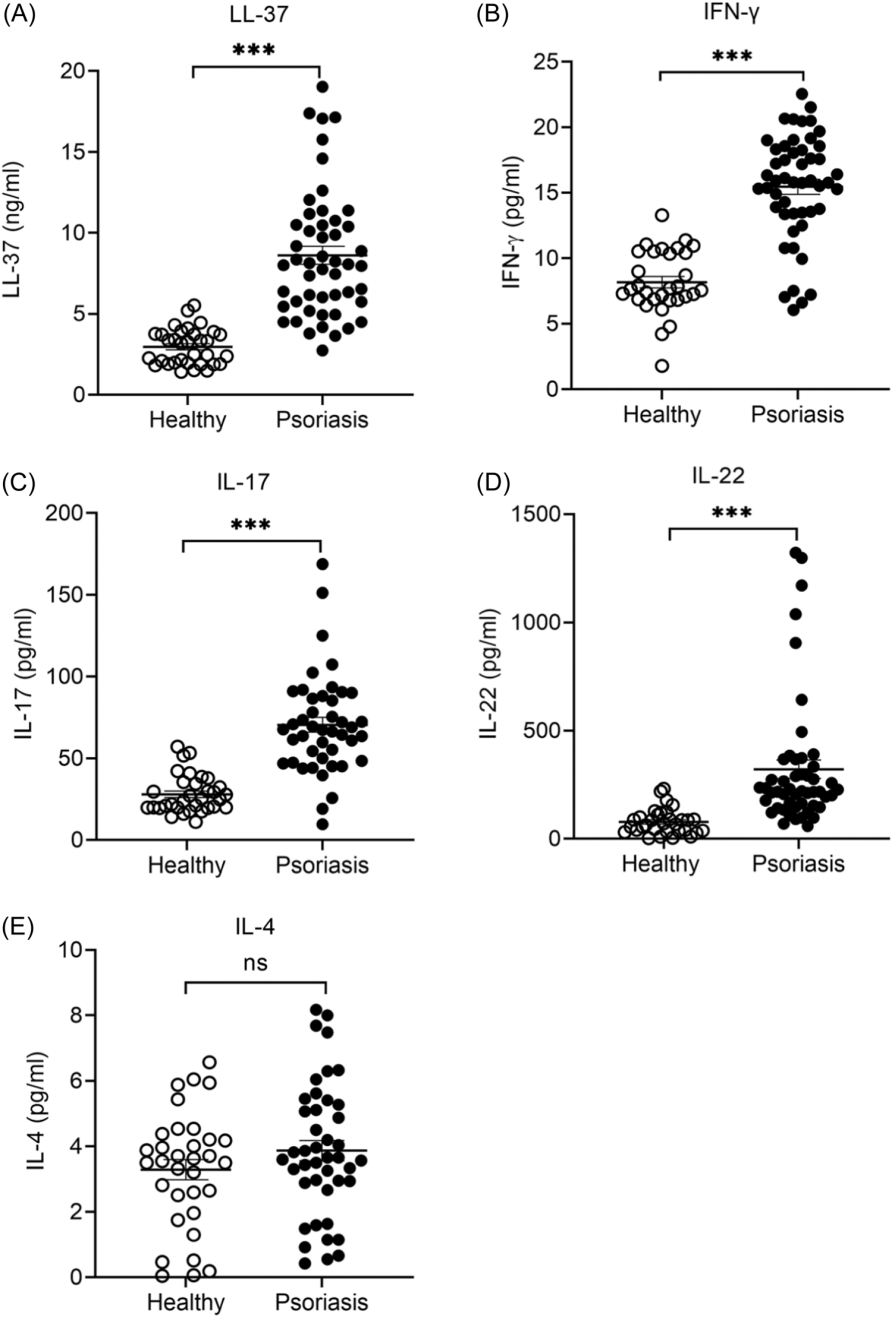
Serum levels of LL‐37 (A) and inflammatory cytokines including IFN‐γ (B), IL‐17 (C), IL‐22 (D), and IL‐4 (E) in PsO patients (*n* = 50) and healthy volunteers (*n* = 33). Comparisons between the two groups were made using an unpaired *t*‐test, with error bars representing the mean ± SEM. ns, not significant, ***p < .001. LL‐37, leucine leucine‐37.

To explore the changes of serological inflammation indicators in PsO, LL‐37, and inflammatory cytokines serum levels were assessed. Serum LL‐37 level in PsO patients (8.62 ± 0.56) was markedly higher than that in healthy donors (2.96 ± 0.19) (Figure 2A). The IFN‐γ, IL‐17, and IL‐22 serum levels in PsO patients were also elevated in comparison with those in healthy control group (healthy, IFN‐γ: 8.17 ± 0.43, IL‐17: 27.94 ± 2.00, IL‐22: 79.12 ± 10.22; PsO, IFN‐γ: 15.46 ± 0.57, IL‐17: 70.50 ± 4.58, IL‐22: 321.4 ± 42.78) (Figure 2B−D). However, the serum concentration of anti‐inflammatory cytokine IL‐4 showed no remarkable difference between the two groups (healthy: 3.29 ± 0.31; PsO: 3.87 ± 0.31) (Figure 2E). These results suggested that the serum LL‐37, IFN‐γ, IL‐17, and IL‐22 levels might be diagnostic biomarkers in PsO.

### 3.3 Correlations between LL‐37 and inflammatory cytokines serum levels in PsO

**Figure 3:**
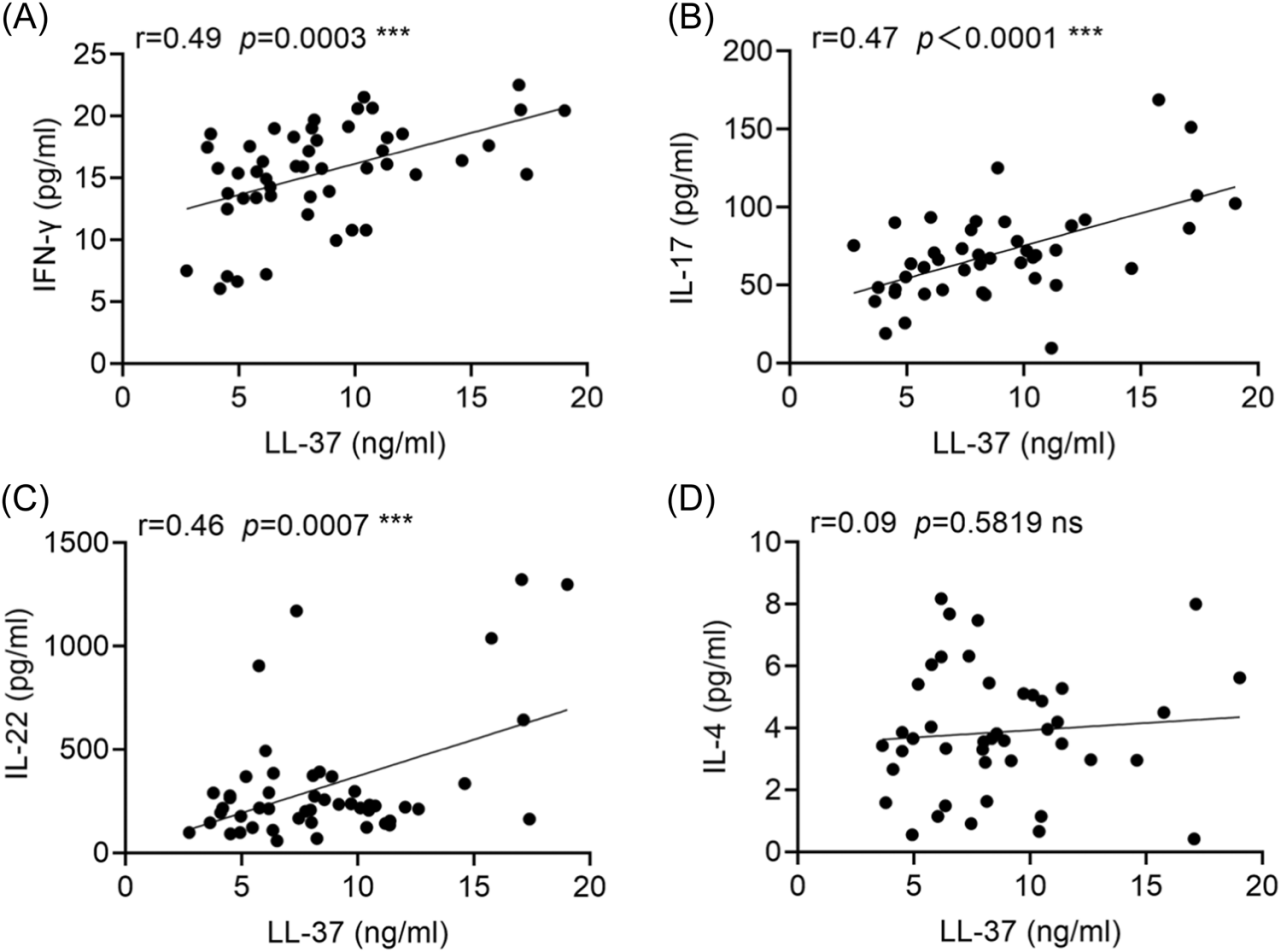
Correlations between serum levels of IFN‐γ (A), IL‐17 (B), IL‐22 (C), IL‐4 (D), and LL‐37 in PsO patients (*n* = 50). The Pearson's correlation test and linear regression were used to calculate the linear regression lines. Data are presented as mean ± SEM. ns, not significant, ***p < .001. LL‐37, leucine leucine‐37; r, correlation coefficient.

To investigate whether LL‐37 was involved in the inflammatory response, we next studied the correlations between LL‐37 and inflammatory cytokines serum levels. Serum level of LL‐37 was positively correlated with IFN‐γ, IL‐17, and IL‐22 in PsO patients (*r* = .49, *p* = .0003; *r* = .47, *p* < .0001; *r* = .46, *p* = .0007, respectively) (Figure 3A−C). However, no obvious correlation between serum LL‐37 and IL‐4 levels was found (*r* = .09, *p* > .05) (Figure 3D). These findings suggested that LL‐37 may participate in the regulation of cytokines production that was implicated in Th1, Th17, and Th22 immune responses in PsO.

### 3.4 LL‐37 and proinflammatory cytokines serum levels decreased in PsO patients after treatment

**Figure 4:**
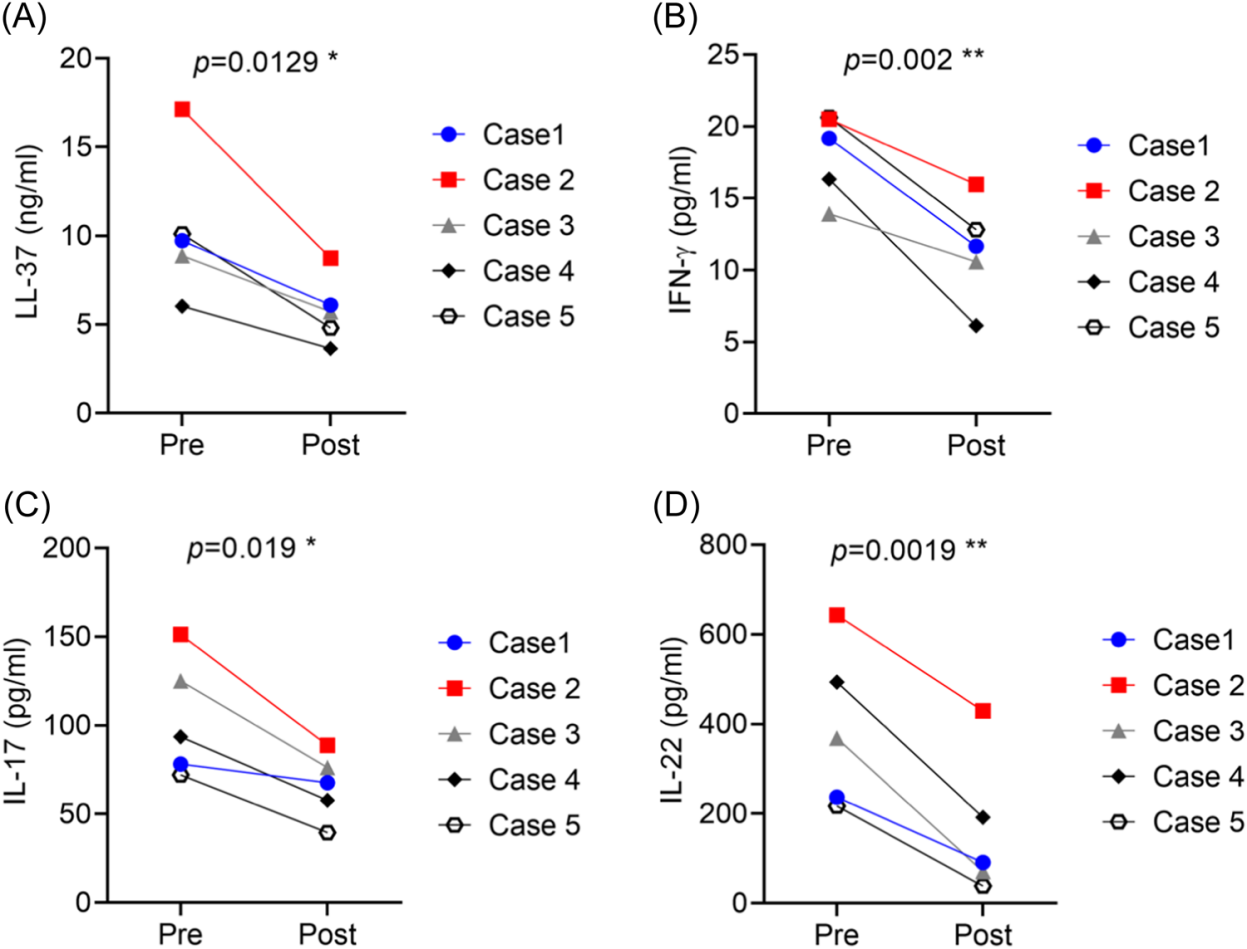
Comparison of serum levels of LL‐37 (A) and inflammatory cytokines including IFN‐γ (B), IL‐17 (C), and IL‐22 (D) in five psoriasis patients before and after treatment. Statistical significance was analyzed by paired *t*‐test. *p < .05, **p < .01. LL‐37, leucine leucine‐37; Post, posttreatment; Pre, pretreatment.

LL‐37 and inflammatory cytokines serum levels were detected in five PsO patients before and after treatment. Posttreatment reduction of LL‐37 was observed (*p* = .0129, paired *t*‐test) (Figure 4A). Similarly, posttreatment patients exhibited lower serum levels of IFN‐γ, IL‐17, and IL‐22 than those before treatment (*p* = .002, *p* = .019, *p* = .0019, respectively) (Figure 4B−D). However, IL‐4 level showed no remarkable difference in these patients before and after treatment (Figure 4E). Our data showed that LL‐37 and proinflammatory cytokines levels decreased in PsO patients after treatment. These results indicated that LL‐37 could reflect the inflammation response, which might act as a promising prognostic biomarker in PsO.

## 4 DISSCUSION

PsO has been regarded as a T‐cell‐dependent autoimmune disorder involving the induction of Th1/Th17 cell responses and abnormal release of inflammatory cytokines.^31^, ^32^ It has been reported that inhibition of spleen tyrosine kinase attenuated PsO‐like inflammation in mice through blockade of dendritic cell‐Th17 inflammation axis.^33^ Targeting IL‐17RC/NF‐κB signaling can reduce hepatic inflammation in PsO patients by preventing dysregulated protein/lipid metabolism.^34^ Our research aimed to investigate inflammation‐related molecules for diagnosis and therapy in PsO. In this study, we observed that serum LL‐37, IFN‐γ, IL‐17, and IL‐22 levels were elevated in PsO patients, and decreased after patients received treatment. Furthermore, LL‐37 level was positively correlated with these proinflammatory cytokines. This study explored the correlations between LL‐37 and inflammatory cytokines, which may provide us some novel insights on PsO pathogenesis.

LL‐37 was elevated in skin lesions of PsO patients and had been recognized as a vital factor for inflammation in PsO.^35^ Our previous data illustrated that serum level of LL‐37 was markedly increased in PsO patients and LL‐37 autoantibodies were correlated with the PASI.^24^ Consistent with that, serum LL‐37 level was elevated in PsO patients in this study. Similar tendency was found in the serum IFN‐γ, IL‐17, and IL‐22 levels, but not IL‐4. LL‐37 was identified as an T cells autoantigen in PsO, and IFN‐γ and Th17 cytokines were secreted by LL‐37‐specific T cells in skin and blood.^36^ On the other hand, the production of LL‐37 could be increased by inflammatory cytokines IFN‐γ, TNF‐α, IL‐17, and IL‐22, whereas it was decreased by anti‐inflammatory cytokines such as IL‐4, IL‐13, and IL‐10.^29^ In this study, we supposed that LL‐37 mainly regulated Th1, Th17, and Th22 pathways but not Th2 response in PsO pathogenesis. Previous studies have illustrated that LL‐37 inhibited DCs activation through TLR ligands, which further prevented T cell activation. The impacts of LL‐37 on T cells cytokines production are complicated, and the mechanism by which LL‐37 regulates PsO pathogenesis merits further investigation.

The correlation between LL‐37 and cytokines levels may indicate the balance between the specified cytokine and LL‐37. This study reported obvious positive correlations between serum LL‐37 and IFN‐γ, IL‐17, IL‐22 levels. However, no remarkable correlation was found between LL‐37 and IL‐4. The circulating LL‐37 may have an effect on IFN‐γ, IL‐17, and IL‐22, which are recognized as crucial cytokines in Th1, Th17, and Th22 pathways in PsO. Kryczek et al. reported that IFN‐γ synergized with IL‐17 to promote the antimicrobial and chemotactic protein β‐defensin 2 production by psoriatic keratinocytes.^37^ Previous study has illustrated the positive correlation between serum inflammatory cytokines and patients' PASI scores,^38^, ^39^, ^40^ suggesting that serum cytokines levels may act as vital parameters to monitor the progression of PsO. However, the correlation between serum LL‐37 level and PsO severity needs to be confirmed.

PsO is associated with an overproduction of antimicrobial peptides, inflammatory cytokines, and chemokines.^6^, ^41^ Consistent with previous study,^29^ we found the posttreatment reduction of serum IFN‐γ, IL‐17, and IL‐22 levels in PsO patients, indicating the improvement in disease progression. We also observed that serum LL‐37 level was decreased after treatment. However, whether the inhibition of LL‐37 production improves PsO needs further investigation due to lacking of the relationship between LL‐37 level and PASI scores. Further study should focus on the therapeutic effect of LL‐37 in PsO.

There are some limitations in our study. First, disease activity of PsO patients should be indicated by PASI scores. In addition, a larger sample of patients should be included to conduct comparison of cytokines and LL‐37 levels in serum pre‐ and posttreatment. Moreover, our results support that LL‐37 may be implicated in inflammation in PsO, however, the mechanisms by which LL‐37 regulates inflammation in PsO require further investigation. Animal and cell experiments should be conducted to confirm the hypothesis.

In summary, we demonstrated that LL‐37 level was positively correlated with IFN‐γ, IL‐17, and IL‐22 levels in serum. Circulating LL‐37 might regulate the production of IFN‐γ, IL‐17, and IL‐22 in PsO. The molecular mechanism and the therapeutic effect of LL‐37 in PsO merit in‐depth investigation.

## 5 CONCLUSION

This study determined that LL‐37 expression was significantly associated with inflammatory response, which may provide us new ideas for diagnosing and monitoring disease activity of PsO. Further detailed studies are required to clarify the role of Ll‐37 in the diagnosis and treatment of PsO.

## AUTHOR CONTRIBUTIONS

Yulin Yuan initiated and designed the research. Yulin Yuan and Juanfeng performed the research and wrote the original draft. Zhi Xie and Qunshi Qin prepared samples, performed data analysis and interpretation. Ru Qin and Shangyang Li contributed to essential reagents and tools and supervised the study. All authors contributed to writing, reviewing, and editing the manuscript. All authors have read and approved the final version of the manuscript.

## CONFLICT OF INTEREST STATEMENT

The authors declare no conflict of interest.

## ETHICS STATEMENT

This study was approved by the ethics committee of The People's Hospital of Guangxi Zhuang Autonomous Region (Ethics No. KY‐KJT‐2021‐83). Informed written consents were obtained from all recruiters.

## Supporting information

Supporting Information.

## Data Availability

The data that support the findings of this study are available from the corresponding author upon reasonable request.
