# Supplementary material for: Serum LL‐37 and inflammatory cytokines levels in psoriasis
Source: Immun Inflamm Dis. 2023 Mar 14;11(3):e802. doi: 10.1002/iid3.802 (PMC10013411; doi:10.1002/iid3.802)
Supplement: Supplementary file 1 — Supporting Information. [file IID3-11-e802-s001.docx]

**Supplementary table 1. Characteristics and blood parameters of healthy donors and patients with psoriasis**

|  | **Healthy** | **psoriasis** | ***p* value^*^** |
| --- | --- | --- | --- |
| Sample size (no.) | 33 | 50 | N/A |
| Mean age (years) | 42 | 46 | NS |
| Sex (M/F) | 22/11 | 34/16 | NS |
| hs-CRP(mg/L) | <0.5 | >5 | N/A |
| Hemoglobin (g/L) | 136 | 131 | NS |
| Platelets (×10^9^/ L) | 277 | 275 | NS |
| WBC (×10^9^/ L) | 8.2 | 6.1 | 0.0002 |
| RBC (×10^12^/ L) | 4.8 | 4.8 | NS |
| Neutrophil (%) | 61.2 | 56.2 | 0.0316 |
| Monocyte (%) | 8.6 | 7.0 | 0.0011 |
| Lymphocyte (%) | 26.7 | 32.2 | 0.0072 |
| Eosinophil (%) | 3.3 | 2.1 | 0.0036 |
| Basophil (%) | 0.6 | 0.6 | NS |

F, Female; M, male; CRP:C-Reactive protein; WBC: White blood cell; NA, not applicable; NS, not significant. Data were shown by mean ± SD or median (25% percentile, 75% percentile).*The level of significance was evaluated by unpaired student *t* test or Mann-Whitney 2-tailed *U* test. *p* value <0.05 was considered statistically significant.
